# The HIV epidemic and human rights violations in Brazil

**DOI:** 10.7448/IAS.16.1.18817

**Published:** 2013-11-12

**Authors:** Monica Malta, Chris Beyrer

**Affiliations:** 1 Social Science Department, ENSP/FIOCRUZ, Rio de Janeiro, Brazil; 2 Department of Epidemiology, Johns Hopkins Bloomberg School of Public Health, Baltimore, MD, USA

Efforts to mitigate stigma and discrimination have been central to the national response to HIV in Brazil, a country historically recognized as a leader in human rights-based HIV prevention, treatment and care [1–3]. Brazil is credited with avoiding a potentially generalized HIV epidemic by providing universal access to antiretroviral therapy (ART) and associated HIV care since 1996 [4]. The Brazilian AIDS policy, although hailed as a model worldwide, has been more difficult to implement among more vulnerable groups of people living with HIV/AIDS, such as drug users, homeless persons, people who use drugs and sex workers, who are often stigmatized as noncompliant or difficult to retain in care [5].

In spite of difficulties in implementing universal ART access among more vulnerable groups, political leaders have implemented diverse strategies aiming at securing universal access to antiretroviral (ARV). The country has produced generic ART since the early 1990s. But the rising costs of second-line treatments prompted the Brazilian government to explore additional strategies. Since 2000, the government made a few public threats to issue a compulsory license for local production of generic versions of patented ARVs, leading several multinational pharmaceutical companies to slash the prices of AIDS medicines. Brazil was also the first country to grant a compulsory license for an AIDS drug – Efavirenz, the ARV drug most used by Brazilian patients. Since this compulsory license was granted, the government has been able to save US$103 million (2007–2011). Today, of the 38 presentations of ARV drugs used for AIDS treatment, 14 are produced domestically [3, 6].

Through the local manufacturing of ART and an emphasis on comprehensive medical care extending beyond HIV-related treatment, the country has reduced mortality rates by 50% and AIDS-related hospitalizations by 80% [4, 7]. This decreased reliance on more expensive emergency-based HIV care has positioned the Brazilian system of HIV treatment as a financially viable model for other low- and middle-income countries [1, 4, 8, 9]. Importantly, the formation of national HIV policy corresponded to a period of social mobilization and democratization across the country, prompting public investment in social services and support systems for people living with HIV (PLHIV) [1, 3, 10]. Brazil's rights-based approach has been documented to extend to historically marginalized populations living with HIV through active partnerships between government and civil society [1, 3, 4, 11, 12].

Historically, the Brazilian response to HIV has supported the reduction of stigma and discrimination among marginalized groups, including both drug users and sex workers [9, 13]. However, recently the political and financial commitment of the National AIDS Program has been vigorously questioned by a social movement initiated in mid-2012, called “What Keeps Us Awake?” and led by researchers, health professionals, activists and local governments (http://oquenostiraosono.tumblr.com/home). The movement was recently presented at the 2nd International HIV Social Sciences and Humanities Conference [14], and it has mobilized society, media, government and many other groups to discuss human rights in the field of HIV/AIDS in Brazil. Activists and researchers in the field of sex workers have also been proactive in highlighting the setbacks observed in preventive initiatives targeting this group in Brazil (http://www.akissforgabriela.com) [12].

In 2012, the Brazilian Ministry of Health censored a campaign targeting young gay men, the group with the highest HIV incidence rates in the country. In March 2013, a series of comics themed on sexuality and education for students was censored before its distribution in Brazilian middle and high public schools. The comic books addressed issues such as adolescence, gender, sexual diversity, sexual and reproductive rights and living with HIV. Those comics were part of the Health and Prevention in Schools project, an initiative of the Ministries of Health and Education in partnership with the Joint United Nations Programme on HIV/AIDS (UNAIDS), United Nations Educational, Scientific and Cultural Organization (UNESCO), United Nations Population Fund (UNFPA) and United Nations Children's Fund (UNICEF).

And on June 4, Brazilian Minister of Health Alexandre Padilha ordered that a poster reading, “I'm happy being a prostitute,” be removed from the Department of STD/AIDS and Viral Hepatitis' website. The poster was one element of a larger campaign entitled Without Shame to Use Condoms, and it was launched on International Prostitutes Day (June 2). The campaign materials were developed by sex workers during a workshop held in March 2013 that was organized by the Department of STD/AIDS and Viral Hepatitis. After the minister's initial decision to remove the “I'm happy being a prostitute” poster, conservative Evangelical groups in Congress mobilized and questioned the whole campaign, making discriminatory and stigmatizing comments and demanding an explanation from the government. By the end of the day, the head of Brazil's Department of STD/AIDS and Viral Hepatitis, Dirceu Greco, had been removed from his position, which was followed by the resignation of his two deputy directors. Following the firing of Greco, on June 17, all civil society organizations resigned from the National AIDS Commission.

According to Nilce Machado, an activist, a sex worker and the president of a sex workers’ NGO who was one of the campaign's faces, “The decision negates the rights of sex workers to be proud of their work, to speak for themselves and to have access to the kind of health information based on citizenship principles that the Brazilian government itself has championed in the past.”

During the Dilma administration, despite strong rhetorical commitment to recognizing the link between human rights and HIV vulnerability, funding and support for rights-based interventions that explicitly target stigma and discrimination, human rights violations and structural impediments against more vulnerable groups such as sex workers have been declining in Brazil.

This conservative path is also being seen in Brazilian changes to drug policy. The Federal Drug Law Bill is still under evaluation in the Brazilian Senate, but since 2013 local governments have been enforcing a policy of compulsory treatment of all drug users: not only adults but also adolescents and children. The government also allocated a large amount of federal funds to therapeutic communities, the majority of them run by conservative, faith-based groups [15].

This was a highly controversial decision supported by the Dilma administration. In 2011, 68 therapeutic communities from all Brazilian regions were visited by a task force led by the Brazilian National Human Rights Commission and the Federal Psychology Council. The group identified a wide range of human rights abuses, including violence and torture; cruel, inhuman and degrading treatment; forced labour and lack of proper medical treatment (including during abstinence crises), among other severe problems. Almost all institutions received government financial support [16]. In this specific arena, Brazil seems to be going against the tide, as opposed to East and Southeast Asia, where compulsory drug detention has been a major problem, and where several governments are announcing reforms [17].

Compulsory addiction treatment is condemned by major international institutions [18]. In 2012, 12 UN agencies issued a joint statement calling for the immediate closure of drug detention centres. According to the statement, “The UN entities which have signed on to this statement call on States that operate compulsory drug detention and rehabilitation centres to close them without delay and to release the individuals detained … and implement voluntary, evidence-informed and rights-based health and social services in the community” [19].

For 30 years of the HIV epidemic, the Brazilian response has been accompanied by vocal, and sometimes contentious, advocacy from scientists, activists and even government officials. Those diverse social actors have been working together and were able to create a paradigm shift in how HIV policies and programmes were developed, prioritized, funded and implemented in the country. The complexity of the response towards the HIV epidemic in Brazil is far from reaching 100% universal access, as pointed out by several studies [5, 7, 20], with stigma and late entry into care being key problems to be addressed [21–23]. In spite of those problems, it is undeniable that Brazil's unprecedented accomplishments in HIV treatment have profoundly influenced global HIV and health policy [3].

However, during the current administration we are seeing marked retrenchments in Brazil's rights-based response. Since the beginning of the epidemic, guaranteeing human rights has been an essential aspect of successful Brazilian strategies regarding the HIV epidemic. It is time, once again, for human rights activists, affected communities, local leaders and researchers to work together in order to turn this tide. The appointment of Dr Fabio Mesquita as head of Brazil's Department of STD/AIDS and Viral Hepatitis (in July 2013) was received with optimism. Mesquita was a pioneer in implementing harm reduction strategies in Brazil in the late 1980s and has been actively engaged in the implementation of evidence-based policy and interventions in Brazil and elsewhere. The impacts of his leadership remain to be seen in the near future. It is critical that he succeeds.
